# Comparative transcriptome and potential antiviral signaling pathways analysis of the gills in the red swamp crayfish, *Procambarus clarkii* infected with White Spot Syndrome Virus (WSSV)

**DOI:** 10.1590/1678-4685-GMB-2016-0133

**Published:** 2017-02-20

**Authors:** Zhi-Qiang Du, Yan-Hui Jin

**Affiliations:** School of Life Science and Technology, Inner Mongolia University of Science and Technology, Baotou, Inner Mongolia Autonomous Region, China

**Keywords:** Procambarus clarkii, WSSV, gills, Illumina sequencing, *de novo* assembly, comparative transcriptomics

## Abstract

Red swamp crayfish is an important model organism for research of the invertebrate innate immunity mechanism. Its excellent disease resistance against bacteria, fungi, and viruses is well-known. However, the antiviral mechanisms of crayfish remain unclear. In this study, we obtained high-quality sequence reads from normal and white spot syndrome virus (WSSV)-challenged crayfish gills. For group normal (GN), 39,390,280 high-quality clean reads were randomly assembled to produce 172,591 contigs; whereas, 34,011,488 high-quality clean reads were randomly assembled to produce 182,176 contigs for group WSSV-challenged (GW). After GO annotations analysis, a total of 35,539 (90.01%), 14,931 (37.82%), 28,221 (71.48%), 25,290 (64.05%), 15,595 (39.50%), and 13,848 (35.07%) unigenes had significant matches with sequences in the Nr, Nt, Swiss-Prot, KEGG, COG and GO databases, respectively. Through the comparative analysis between GN and GW, 12,868 genes were identified as differentially up-regulated DEGs, and 9,194 genes were identified as differentially down-regulated DEGs. Ultimately, these DEGs were mapped into different signaling pathways, including three important signaling pathways related to innate immunity responses. These results could provide new insights into crayfish antiviral immunity mechanism.

## Introduction

Unlike vertebrates, invertebrates lack an acquired immune system, but they develop the innate immune system, including cellular and humoral immune responses (Du *et al.*, 2010). When hosts suffer insults or infections from pathogens, these genes can be synergistically mobilized to play their respective roles in cellular defense, especially in the humoral immune response (Taffoni and Pujol, 2015). Further study of the coordination mechanisms of the invertebrate innate immune system by immune-related genes is crucial.

As a typical invertebrate, the red swamp crayfish is used as a model organism to research the response principles of the invertebrate innate immune system. This species is native to Northeastern Mexico and South America and was introduced into China from Japan in the 1930s (Shen *et al.*, 2014). Because of its good fitness characteristics, strong adaptability to a changing environment, and high fecundity, the red swamp crayfish has been widely aquacultured in China (Manfrin *et al.*, 2015). Currently, this crayfish has become one of the economically most important aquacultured species. Additionally, the excellent resistances of red swamp crayfish against bacteria, fungi, and viruses are well-known. Recent studies have shown that antibacterial and antifungal mechanisms, such as the Toll and Imd pathways, are strongly conserved (Ermolaeva and Schumacher, 2014). However, the antiviral mechanism in this species remains unclear (Ramos and Fernandez-Sesma, 2015). Systematically identifying the antiviral genes and antivirus-related signaling pathways in this species through transcriptome sequencing is crucial.

Recently, several reports have described transcriptome sequencing results of crayfish tissues, including the hepatopancreas, muscle, ovary, testis, eyestalk, spermary, epidermis, branchia, intestines, and stomach (Shen *et al.*, 2014; Manfrin *et al.*, 2015). However, the transcriptome of crayfish gills or white spot syndrome virus (WSSV)-challenged tissues have not been reported. More importantly, the crayfish gills are in direct contact with the external environment, and gill cells are crucial in the response to external biotic and abiotic factors, especially pathogenic microorganisms (Clavero-Salas *et al.*, 2007). Due to their large number of hemocytes, gills play an important role in cellular host defense against pathogens (Egas *et al.*, 2012). The gills are a vital organ that can remove invasive pathogens through an efficient and specific immune response. The study of the gill transcriptome will define an important part of the research field of the innate immune response mechanism.

Next-generation sequencing (NGS) technology has been widely used to explore and uncover vast genetic information in model organisms (Jiang *et al.*, 2014). NGS technology is superior to the traditional Sanger sequencing technology in many aspects. For example, NGS technology can provide enormous amounts of sequence data in much shorter times and at a much lower cost (Martin and Wang, 2011). The expressed sequences produced using NGS technologies are usually 10-to100-fold greater than the number identified by traditional Sanger sequencing technologies (Christie *et al.*, 2015). In this study, the HiSeq sequencing technology was used to sequence the transcriptomes of normal and WSSV-challenged crayfish gills. This approach was used to generate expression profiles and to discover differentially expressed genes (DEGs) between normal and WSSV-challenged crayfish gills. The functions of DEGs were annotated and classified by the Gene Ontology (GO), Cluster of Orthologous Groups (COG), and Kyoto Encyclopedia of Genes and Genomes (KEGG) databases. We believe the data obtained from this study could provide an important resource for research about genes functions, molecular events, and signaling pathways related to the invertebrate antiviral immune response.

## Materials and Methods

### Preparation of crayfish tissues and immune challenge

*P. clarkii* (weighing approximately 15–20 g) were purchased from an aquaculture commercial market in Hangzhou, Zhejiang Province, China. The collected crayfish were originally cultured in water tanks at 26–28°C for 10 days and fed twice a day with artificial food throughout the whole experiment (Li Y *et al.*, 2012). To mimic WSSV infection, WSSV (3.2 10^7^ copies per crayfish) was injected into the abdominal segment of each crayfish (Du *et al.*, 2009). Thirty-six hours after the viral challenge, gills were collected from at least ten WSSV-challenged crayfish (Group WSSV-challenged (GW)). Gills were also collected from ten controls, uninfected crayfish designated as the Group normal (GN). All gills were immediately frozen in liquid nitrogen after collection, and samples were temporarily stored at −80°C until total RNA extraction (Du and Jin, 2015).

### RNA isolation and Illumina sequencing

The two types of gill tissue samples (GN and GW) previously frozen in liquid nitrogen were delivered to the Beijing Genomics Institute-Shenzhen (BGI, Shenzhen, China) for total RNA extraction. In brief, total RNA from crayfish gills was extracted with TRIzol reagent in accordance with manufacturer's protocol (Invitrogen, Life Technologies, Grand Island, NY, USA). The quality of RNA samples treated with DNase I (Invitrogen) was examined for subsequent procedures, including mRNA purification, cDNA library construction and transcriptome sequencing. Approximately 5 μg of DNase-treated total RNA was used to construct a cDNA library following the protocols of the Illumina TruSeq RNA Sample Preparation Kit (Illumina, San Diego, CA, USA). After necessary quantification and qualification, the library was sequenced using an Illumina HiSeq 2000 instrument with 100 bp paired-end (PE) reads for both groups.

### Transcriptome *de novo* assembly and analysis

Transcriptome *de novo* assembly for all samples was carried out by the RNA-Seq *de novo* assembly program Trinity (Dedeine *et al.*, 2015). In brief, raw reads generated by the Illumina HiSeq 2000 sequencer were originally trimmed by removing the adapter sequences. After low-quality reads with quality scores ≤ 20 and short reads with a length ≤ 10 bp were removed, high-quality clean reads were obtained to execute transcriptome *de novo* assembly using the Trinity software with the default parameters. Generally, three steps were performed, including Inchworm, Chrysalis and Butterfly (He *et al.*, 2015). Initially, high-quality clean reads were processed by Inchworm to form longer fragments, called contigs. Then, contigs were connected by Chrysalis to obtain unigenes, which could not be extended on either end. Unigenes resulted in de Bruijn graphs. Finally, the de Bruijn graphs were processed by Butterfly to obtain transcripts (Li *et al.*, 2013).

### Transcriptome annotation and Gene Ontology analysis

After transcriptome *de novo* assembly was finished, transcripts were used to carry out annotation tasks, including protein functional annotation, COG functional annotation, GO functional annotation, and pathways annotation. These tasks were based on sequence similarity with known genes. In detail, assembled contigs were annotated with sequences available in the NCBI database, using the BLASTx and BLASTn algorithms (Leng *et al.*, 2015). The unigenes were aligned by a BLASTx search against NCBI protein databases, including the non-redundant (Nr) sequence, Swiss-Prot, KEGG, and COG databases (Li *et al.*, 2015). However, none of the BLASTx hits were aligned by a BLASTn search against the NCBI non-redundant nucleotide database (Nt). The above alignments were executed to establish the homology of sequences with known genes (with a cutoff E-value ≤ 10^-5^) (Li ST *et al.*, 2012). Then, the best alignment results were used to decide the sequence orientation and the protein coding region prediction (CDS) of the unigenes. Functional annotation was executed with Gene Ontology (GO) terms (www.geneontology.org) that were analyzed using the Blast2GO software (http://www.blast2go.com/b2ghome) (Conesa *et al.*, 2005). Based on the KEGG database, the complex biological behavior of the genes was analyzed through pathway annotation.

### Identification of differentially expressed genes

To acquire the expression profiles of transcripts in crayfish gills, cleaned reads were first mapped to all transcripts using Bowtie software (Langmead and Salzberg, 2012). Then, DEGs were obtained on the basis of fragments per kilobase of exon per million fragments mapped (FPKM) of the genes, followed by a False Discovery Rate (FDR) control, to correct for the P-value (Mortazavi *et al.*, 2008). DEGs were identified using EDGER software (empirical analysis of digital gene expression data in R) (Robinson *et al.*, 2010). In the analysis process, the filtering threshold was set as a 0.5 FDR control. Ultimately, an FDR ≤ 0.001 and the absolute value of the log2Ratio ≥ 1 were used as the filtering threshold to determine the significance of DEGs (Banani *et al.*, 2016). DEGs (GW *vs* GN) were identified through a comparative analysis of the above data.

### Quantitative real-time PCR

Quantitative real-time PCR (qRT-PCR) methods were used to determine the RNA levels for 15 selected genes related to innate immune response (Wang *et al.*, 2015). For the qRT-PCR analysis, cDNA templates from the samples were diluted 20-fold in nuclease-free water and used as templates for the PCR reaction. Gene-specific primers sequences were designed using Primer Premier 6 software on the basis of each identified gene sequence from the transcriptome library (Lu *et al.*, 2014). The specific primers*Pc-18 S RNA*-qRT-F (5'-tct tct tag agg gat tag cgg-3') and *Pc-18 S RNA*-qRT-R (5'-aag ggg att gaa cgg gtt a-3') were used to amplify the *18 S RNA* gene as an internal control. qRT-PCR was performed following the manufacturer's instructions of SYBR Premix Ex Taq (Takara, Dalian, China) using a real-time thermal cycler (Bio-Rad, USA) in a total volume of 10 μl containing 5 μl of 2 Premix Ex Taq, 1 μl of the 1:20 diluted cDNA, and 2 μl (1 μM) each of the forward and reverse primers. The amplification procedure was comprised of an initial denaturation step at 95°C for 3min, followed by 40 cycles of 95°C for 15s, 58°C for 40 s, and melting from 65°C to 95°C. Three parallel experiments were performed (Du *et al.*, 2015). Furthermore, the differentially expressed levels of the target genes between the groups were calculated by the 2^-ΔΔCT^ analysis method (Livak and Schmittgen, 2001). Data obtained were subjected to a statistical analysis, followed by an unpaired sample *t*-test. A significant difference was assigned when P < 0.05 or P < 0.01.

## Results

### Illumina sequencing of the crayfish gills transcriptome

After the GN sample was cleaned and quality-tested, 39,390,280 clean reads were identified from 40,384,330 raw reads, which corresponded to 3,939,028,000 total nucleotides (nt). The Q20 percentage (percentage of bases whose quality was ≥ 20 in the clean reads), N percentage (percentage of uncertain bases after filtering) and GC percentage were 98.02%, 0.00% and 41.52%, respectively (Table 1). For the GW sample, 34,011,488 clean reads were identified from 34,840,334 raw reads, which corresponded to 3,401,148,800 total nucleotides (nt). The Q20 percentage, N percentage and GC percentage were 98.04%, 0.00% and 41.98%, respectively (Table 1). All these sequences were used for further analysis.

**Table 1:** Summary of Illumina sequencing output for the GN and the GW.

Sample	Total raw reads	Total clean reads	Total clean nucleotides (nt)	Q20 percentage	N percentage	GC percentage
GN-gills	40,384,330	39,390,280	3,939,028,000	98.02%	0.00%	41.52%
GW-gills	34,840,334	34,011,488	3,401,148,800	98.04%	0.00%	41.98%

### *De novo* assembly of the transcriptome

After the low-quality and short length reads were removed, high-quality clean reads were obtained, and transcriptome *de novo* assembly was performed using Trinity software with its default parameters (Ali *et al.*, 2015). For GN, 39,390,280 high-quality clean reads were randomly assembled to produce 172,591 contigs with an N50 of 690 bp. The contigs were further assembled and clustered into 94,479 unigenes with a mean length of 644 nt, which were composed of 10,931 distinct clusters and 83,548 distinct singletons. The N50 of the above unigenes was 1,345 bp (Table 2). For GW, 34,011,488 high-quality clean reads were randomly assembled to produce 182,176 contigs with an N50 of 528 bp. The contigs were further assembled and clustered into 95,959 unigenes with a mean length of 558 nt, which were composed of 8,299 distinct clusters and 87,660 distinct singletons. The N50 of the above unigenes was 1,007 bp (Table 2).

**Table 2:** Summary of the assembly analysis for GN and GW.

Dataset name	Group normal (GN)		Group WSSW-challenged (GW)	
	Contigs	Unigenes	Contigs	Unigenes
Total number	172,591	94,479	182,176	95,959
Total length (nt)	60,746,050	60,823,435	57,529,732	53,499,561
Mean length (nt)	352	644	316	558
N50	690	1,345	528	1,007
Total consensus sequences	-	94,479	-	95,959
Distinct clusters	-	10,931	-	8,229
Distinct singletons	-	83,548	-	87,660

For GN, the lengths of unigenes were primarily distributed between 200 nt and 2,000 nt. The percentage of unigenes with lengths from 201–500 bp was 70.30% (66,423), from 501–1,000 bp was 14.07% (13,294), from 1,001–1,500 bp was 5.40% (5,099), from 1,501–2,000 bp was 3.23% (3,048), and > 2,000 bp was 7.00% (6,615). For GW, the percentage of unigenes with lengths from 201–500 bp was 74.12% (71,124), from 501–1,000 bp was 13.44% (12,897), from 1,001–1,500 bp was 4.91% (4,711), from 1,501–2,000 bp was 2.58% (2,479), and > 2,000 bp was 4.95% (4,748).

### Functional annotation of predicted proteins

Transcripts were combined with data from two other transcriptomes from Mousavi *et al.* (2014). First, sequence annotation was carried out on the basis of unigenes from the merged group (Garcia-Seco *et al.*, 2015). Then, the putative functions of all unigenes were analyzed based on GO and COG classifications (Young *et al.*, 2010). In the present study, based on the merged group transcriptome data (98,676 unigenes), a basic sequence analysis was performed to understand the functions of the crayfish gill transcriptome before the analysis of DEGs related to WSSV infection. Among the predicted sequences, a total of 39,482 unigene sequences were annotated using a BLASTx alignment with an E-value ≤ 10^-5^. A total of 35,539 (90.01%), 14,931 (37.82%), 28,221 (71.48%), 25,290 (64.05%), 15,595 (39.50%), and 13,848 (35.07%) unigenes had significant matches with sequences in the Nr, Nt, Swiss-Prot, KEGG, COG and GO databases, respectively. In brief, a total of 35,539 transcripts (90.01% of all annotated transcripts) had significant hits in the Nr protein database. The gene names of the top BLAST hits were assigned to each transcript with significant hits. Among them, 2,682 (7.55%) transcripts were matched with genes from *Paramecium tetraurelia*, 2,362 (6.65%) transcripts were matched with genes from *Daphnia pulex*; 3,265 (9.19%) transcripts were matched with genes from *Tetrahymena thermophila*; 1,302 (3.66%) transcripts were matched with genes from *Tribolium castaneum*; 1,292 (3.64%) transcripts were matched with genes from *Ichthyophthirius multifiliis*; and 929 transcripts were matched with genes from *Pediculus humanus*.

**Figure 1:**
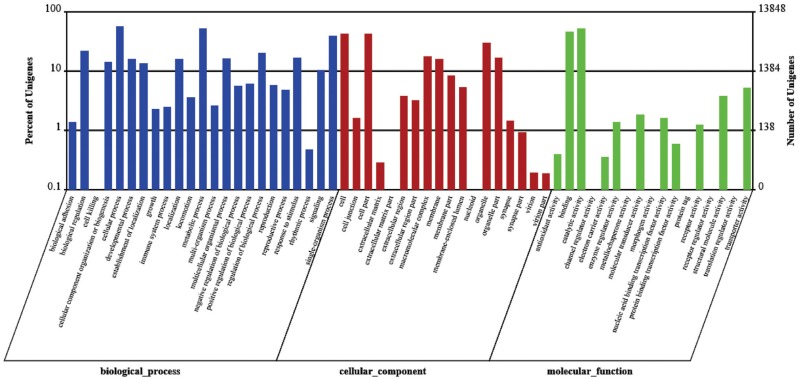
Gene ontology (GO) classification of transcripts from the two gill samples (GN and GW). The three main GO categories include biological process (blue), cellular component (red), and molecular function (green).

The GO classification is an international standardized gene function classification system that provides a dynamically updated controlled vocabulary and an exactly defined conception to describe genes' characteristics and their products in any organism (Di *et al.*, 2015). The GO classification includes three ontologies: biological process, cellular component and molecular function (Chen *et al.*, 2015). In this study, a GO analysis was carried out using Blast2GO software. A total of 13,848 transcripts annotated in the GO database were categorized into 58 functional groups, including the three main GO ontologies (Figure 1). Among these functional groups, “biological regulation”, “cellular process”, “metabolic process”, “cell”, “single-organism process”, “cell part”, “binding”, and “catalytic activity” terms were predominant.

**Figure 2:**
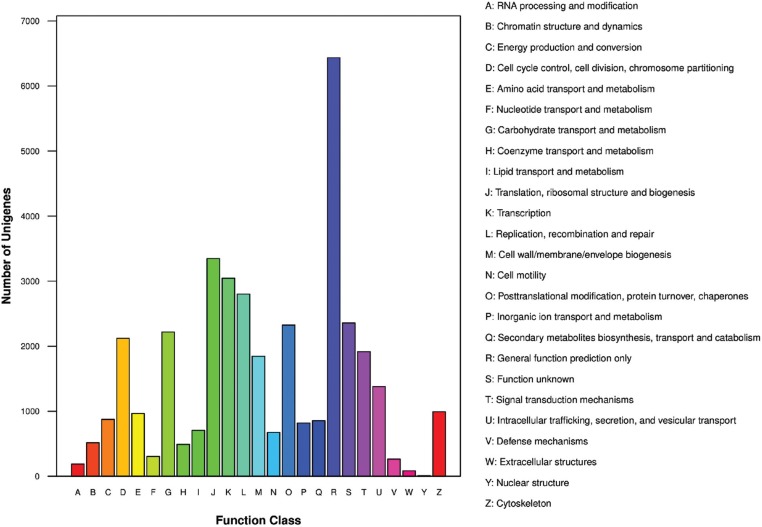
Cluster of orthologous groups (COG) classification of putative proteins.

COGs were delineated by comparing protein sequences encoded in complete genomes, representing major phylogenetic lineages (Tatusov *et al.*, 2003). COG classification analysis is also an important method for unigene functional annotation and evolutionary research (Lai and Lin, 2013). We used the COG classification to further evaluate the completeness of the transcriptome library and the effectiveness of the annotation methods. A total of 15,959 unigenes were mapped to 25 different COG categories. In these 25 different categories, the largest COG group was category R, representing “general function prediction only” (6,435 unigenes, 41.26%), followed by category J, representing “translation, ribosomal structure and biogenesis” (3,347 unigenes, 21.46%); category K, representing “transcription” (3,044 unigenes, 19.52%); and category L, representing “replication, recombination and repair” (2,802 unigenes, 17.97%; Figure 2).

KEGG is a bioinformatics resource for linking genomes to life and the environment (Mao *et al.*, 2005). The KEGG pathway database records networks of molecular interactions in cells and variants specific to particular organisms (Chen *et al.*, 2015). The genes from the merged groups (GN and GW) were categorized using the KEGG database to obtain more information to predict the unigene functions (Feng *et al.*, 2015). A total of 25,290 unigenes were classified into 257 KEGG pathways. Among these KEGG pathways, the top 30 statistically significant KEGG classifications are shown in Table 3. Moreover, some important innate immunity-related pathways were predicted in this KEGG database, including *Vibrio cholerae* infection (1092 sequences, 4.32%), focal adhesion (910 sequences, 3.6%), Epstein-Barr virus infection (860 sequences, 3.40%), lysosome (610 sequences, 2.41%), HTLV-I infection (596 sequences, 2.36%), Herpes simplex infection (593 sequences, 2.34%), salmonella infection (576 sequences, 2.28%), MAPK signaling pathway (542 sequences, 2.14%), adherens junction (467 sequences, 1.85%), and so on (Table 3). Notably, the insulin signaling pathway, Wnt signaling pathway, mRNA surveillance pathway, endocytosis, phagosome, and tight junction functions were present in the top 40 statistically significant KEGG classifications.

**Table 3:** Top 30 statistically significant KEGG classifications.

No.	Pathway	Pathway definition	Number of sequences
1	path: ko01100	Metabolic pathways	3371 (13.33%)
2	path: ko05146	Amoebiasis	1148 (4.54%)
3	path: ko05110	*Vibrio cholerae* infection	1092 (4.32%)
4	path: ko05016	Huntington's disease	973 (3.85%)
5	path: ko04810	Regulation of actin cytoskeleton	950 (3.76%)
6	path: ko04510	Focal adhesion	910 (3.6%)
7	path: ko03040	Spliceosome	894 (3.53%)
8	path: ko05200	Pathways in cancer	879 (3.48%)
9	path: ko05169	Epstein-Barr virus infection	860 (3.4%)
10	path: ko03013	RNA transport	847 (3.35%)
11	path: ko00230	Purine metabolism	781 (3.09%)
12	path: ko04145	Phagosome	643 (2.54%)
13	path: ko04144	Endocytosis	638 (2.52%)
14	path: ko04270	Vascular smooth muscle contraction	633 (2.5%)
15	path: ko03010	Ribosome	632 (2.5%)
16	path: ko04530	Tight junction	616 (2.44%)
17	path: ko00240	Pyrimidine metabolism	616 (2.44%)
18	path: ko04142	Lysosome	610 (2.41%)
19	path: ko04141	Protein processing in endoplasmic reticulum	607 (2.4%)
20	path: ko05166	HTLV-I infection	596 (2.36%)
21	path: ko05168	Herpes simplex infection	593 (2.34%)
22	path: ko05132	Salmonella infection	576 (2.28%)
23	path: ko03015	mRNA surveillance pathway	563 (2.23%)
24	path: ko04120	Ubiquitin-mediated proteolysis	557 (2.2%)
25	path: ko04010	MAPK signaling pathway	542 (2.14%)
26	path: ko04020	Calcium signaling pathway	538 (2.13%)
27	path: ko05414	Dilated cardiomyopathy	524 (2.07%)
28	path: ko05164	Influenza A	517 (2.04%)
29	path: ko05410	Hypertrophic cardiomyopathy (HCM)	495 (1.96%)
30	path: ko04970	Salivary secretion	493 (1.95%)

### Differentially expressed genes analysis of crayfish gills after WSSV infection

**Figure 3:**
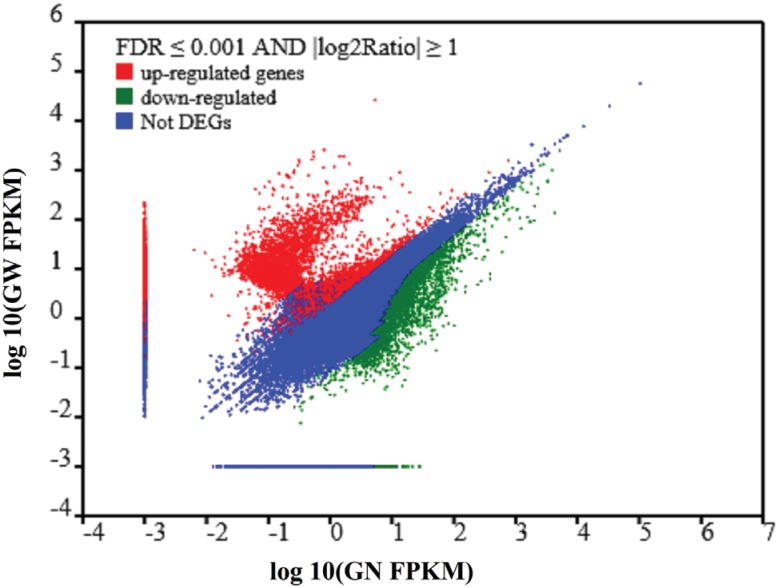
Comparative analysis of the gene expression levels for two transcript libraries between normal (GN) and WSSV-challenged (GW) crayfish gills. Red dots represent transcripts significantly up-regulated in GW, while green dots represent significantly down-regulated transcripts. The parameters “FDR ≤ 0.001” and “| log2 Ratio| ≥ 1” were used as the threshold to judge the significance of gene expression differences.

Previous sequence analysis and annotation for all unigenes in the merged group provided some useful information to analyze the crayfish gills transcriptome. Even more importantly, variation in the gene expression level of crayfish gills after WSSV infection was expected. In this study, an FDR ≤ 0.001 and an absolute value of log2Ratio ≥ 1 were used as the filtering threshold to determine up-regulated or down-regulated genes between normal and WSSV-challenged crayfish gills. As shown in Figure 3, a total of 22,062 DEGs with a > two-fold change were identified by the comparative analysis between the GN and GW samples, including 12,868 differentially up-regulated and 9,194 differentially down-regulated genes. In brief, among these 22,062 DEGs, 12,826 genes were expressed in both GN and GW, including 3,632 differentially up-regulated genes and 9,194 differently down-regulated genes. Moreover, 9,236 genes were only expressed in the GW sample.

**Figure 4:**
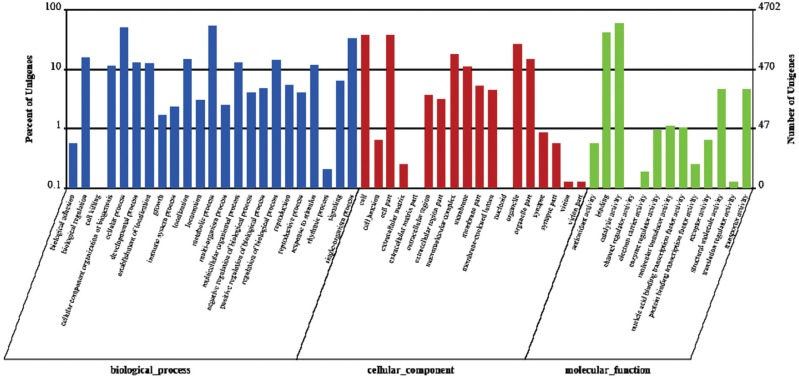
Gene ontology (GO) classification analysis of differentially expressed genes (DEGs) between GN and GW. The three main GO categories include biological process (blue), cellular component (red), and molecular function (green).

To confirm the biological function of DEGs between GN and GW, GO classification and KEGG pathway analysis for the DEGs were carried out (Gupta *et al.*, 2015). A GO classification analysis was conducted on the annotated transcripts using Blast2GO. As shown in Figure 4, a total of 4,702 DEGs were identified after a comparison between GN and GW. Significantly altered expression (up/down) was present for 45 GO terms (P < 0.05) and 32 GO terms, respectively (P < 0.01). Among the former group, there were 15, 8, and 22 GO terms that belonged to “cellular component (C)”, “molecular function (F)” and “biological process (P)”, respectively. Among the latter group, there were 14, 3, and 15 GO terms that belonged to “cellular component (C)”, “molecular function (F)” and “biological process (P)”, respectively. The GO analysis revealed that gene clusters with significant differential expression mainly concentrated in the aspect of biological process.

Next, all DEGs were mapped in the KEGG database to search for genes involved in innate immune response or signaling pathways. A total of 7,918 DEGs were assigned to 256 KEGG pathways. The KEGG pathway analysis identified 100 pathways that were significantly changed (P < 0.05),of which 80 reached a higher level of significance (P < 0.01) in the GW compared with the GN. Some of the significantly altered KEGG pathways were related to the innate immunity response, including apoptosis, *Staphylococcus aureus* infection, lysosome, melanogenesis, TGF-beta signaling pathway, NOD-like receptor signaling pathway, PPAR signaling pathway, focal adhesion, toll-like receptor signaling pathway, and bacterial invasion of epithelial cells (Table 4).

**Table 4:** Top 80 differentially expressed pathways between GW and GN.

No.	Pathway	Number of DEGs	P-value	Pathway ID
1	Glycolysis / Gluconeogenesis	99 (1.25%)	3,78E-199	ko00010
2	RNA transport	219 (2.77%)	4.26E-92	ko03013
3	Epithelial cell signaling	64 (0.81%)	3.64E-19	ko05120
4	Apoptosis	73 (0.92%)	2.29E-17	ko04210
5	Allograft rejection	3 (0.04%)	7.15E-15	ko05330
6	Renin-angiotensin system	31 (0.39%)	2.42E-14	ko04614
7	p53 signaling pathway	55 (0.69%)	1.62E-12	ko04115
8	mTOR signaling pathway	126 (1.59%)	2.34E-12	ko04150
9	Endocytosis	177 (2.24%)	2.35E-11	ko04144
10	*Staphylococcus aureus* infection	36 (0.45%)	1.87E-10	ko05150
11	Leishmaniasis	28 (0.35%)	4.16E-10	ko05140
12	Protein processing in ER	249 (3.14%)	9.51E-10	ko04141
13	Synthesis and degradation of ketone bodies	12 (0.15%)	1.05E-09	ko00072
14	Metabolism of xenobiotics by P450	50 (0.63%)	3.28E-09	ko00980
15	Phe, Tyr and Try biosynthesis	13 (0.16%)	4.22E-09	ko00400
16	Taurine and hypotaurine metabolism	5 (0.06%)	5.99E-09	ko00430
17	Collecting duct acid secretion	44 (0.56%)	9.75E-09	ko04966
18	Chronic myeloid leukemia	43 (0.54%)	2.35E-08	ko05220
19	Pentose phosphate pathway	30 (0.38%)	2.69E-08	ko00030
20	ABC transporters	112 (1.41%)	7.07E-08	ko02010
21	Thyroid cancer	18 (0.23%)	1.21E-07	ko05216
22	Lysosome	268 (3.38%)	2.02E-07	ko04142
23	Morphine addiction	49 (0.62%)	2.35E-07	ko05032
24	Valine, leucine and isoleucine biosynthesis	12 (0.15%)	2.72E-07	ko00290
25	Melanogenesis	108 (1.36%)	5.39E-07	ko04916
26	Butanoate metabolism	40 (0.51%)	5.58E-07	ko00650
27	Base excision repair	45 (0.57%)	6.18E-07	ko03410
28	Long-term depression	41 (0.52%)	6.57E-07	ko04730
29	Hepatitis C	73 (0.92%)	2.47E-06	ko05160
30	Mismatch repair	23 (0.29%)	3.24E-06	ko03430
31	Salivary secretion	185 (2.34%)	3.85E-06	ko04970
32	Histidine metabolism	38 (0.48%)	4.50E-06	ko00340
33	Bacterial invasion of epithelial cells	102 (1.29%)	1.05E-05	ko05100
34	Amoebiasis	458 (5.78%)	1.21E-05	ko05146
35	Propanoate metabolism	72 (0.91%)	1.25E-05	ko00640
36	Synaptic vesicle cycle	85 (1.07%)	1.39E-05	ko04721
37	Ubiquinone and terpenoid-quinone biosynthesis	11 (0.14%)	1.48E-05	ko00130
38	Influenza A	201 (2.54%)	1.78E-05	ko05164
39	Retrograde endocannabinoid signaling	71 (0.9%)	2.46E-05	ko04723
40	Prostate cancer	113 (1.43%)	3.11E-05	ko05215
41	Metabolic pathways	1189 (15.02%)	3.81E-05	ko01100
42	Starch and sucrose metabolism	52 (0.66%)	3.95E-05	ko00500
43	Glyoxylate and dicarboxylate metabolism	50 (0.63%)	7.25E-05	ko00630
44	Cocaine addiction	39 (0.49%)	0.000103	ko05030
45	Endocrine	97 (1.23%)	0.000119	ko04961
46	Phenylalanine metabolism	33 (0.42%)	0.000136	ko00360
47	HTLV-I infection	198 (2.5%)	0.000137	ko05166
48	Chemokine signaling pathway	116 (1.47%)	0.000142	ko04062
49	Vasopressin-regulated water reabsorption	88 (1.11%)	0.000148	ko04962
50	Endometrial cancer	42 (0.53%)	0.00015	ko05213
51	TGF-beta signaling pathway	65 (0.82%)	0.000158	ko04350
52	NOD-like receptor signaling pathway	68 (0.86%)	0.000224	ko04621
53	Regulation of actin cytoskeleton	228 (2.88%)	0.000291	ko04810
54	Transcriptional misregulation in cancer	116 (1.47%)	0.000355	ko05202
55	GnRH signaling pathway	103 (1.3%)	0.000478	ko04912
56	Glycine, serine and threonine metabolism	68 (0.86%)	0.000653	ko00260
57	Citrate cycle (TCA cycle)	89 (1.12%)	0.000814	ko00020
58	Pancreatic cancer	67 (0.85%)	0.000861	ko05212
59	Cardiac muscle contraction	95 (1.2%)	0.000893	ko04260
60	Tyrosine metabolism	66 (0.83%)	0.000972	ko00350
61	Sphingolipid metabolism	31 (0.39%)	0.00143	ko00600
62	Oocyte meiosis	203 (2.56%)	0.001598	ko04114
63	Adipocytokine signaling pathway	68 (0.86%)	0.001695	ko04920
64	Gastric acid secretion	151 (1.91%)	0.001769	ko04971
65	Peroxisome	173 (2.18%)	0.001809	ko04146
66	PPAR signaling pathway	106 (1.34%)	0.001873	ko03320
67	Drug metabolism - other enzymes	44 (0.56%)	0.001887	ko00983
68	Neurotrophin signaling pathway	128 (1.62%)	0.002169	ko04722
69	Tryptophan metabolism	69 (0.87%)	0.002208	ko00380
70	Cysteine and methionine metabolism	45 (0.57%)	0.002622	ko00270
71	Taste transduction	26 (0.33%)	0.003256	ko04742
72	Fatty acid biosynthesis	2 (0.03%)	0.003716	ko00061
73	Focal adhesion	268 (3.38%)	0.004214	ko04510
74	Glycosphingolipid biosynthesis - ganglio series	13 (0.16%)	0.004679	ko00604
75	Huntington's disease	315 (3.98%)	0.005037	ko05016
76	Toll-like receptor signaling pathway	66 (0.83%)	0.00549	ko04620
77	Insect hormone biosynthesis	12 (0.15%)	0.005932	ko00981
78	D-Arginine and D-ornithine metabolism	13 (0.16%)	0.007501	ko00472
79	Progesterone-mediated oocyte maturation	151 (1.91%)	0.007619	ko04914
80	Pancreatic secretion	122 (1.54%)	0.009396	ko04972

### Analysis of transcriptome data by qRT-PCR

We chose 15 genes related to the innate immunity response and evaluated their differential expression level between the GW and the GN, using qRT-PCR (Gao *et al.*, 2015). For these candidate genes, the variation trends of the qRT-PCR expression profiles were found to be consistent with the RNA-seq data (Table 5). There were similar trends in the genes that were up-/down-regulated between the qRT-PCR and the transcriptome data, implying that the differential expression changes identified by the RNA-Seq data were reliable.

**Table 5:** Comparison of relative fold change of RNA-Seq and qRT-PCR results between GW and GN.

Gene name	Protein identity	Fold variation (GW/GN)	
		transcriptome	qRT-PCR
CL3739.Contig3_All	Lysozyme	53.80 (up)	20.51 (up)
Unigene68353_All	Integral membrane protein	42.34 (up)	25.63 (up)
Unigene56169_All	Apoptosis-regulated protein	26.33 (up)	16.77 (up)
CL88.Contig2_All	Serine protease inhibitor	24.41 (up)	18.52 (up)
CL4190.Contig1_All	Chitin binding-like protein	7.29 (up)	16.87 (up)
CL1412.Contig2_All	Interleukin enhancer binding factor	4.83 (up)	2.46 (up)
CL6575.Contig2_All	Anti-lipopolysaccharide factor	4.11 (up)	5.63 (up)
CL818.Contig4_All	Dicer 2	4.09 (up)	3.48 (up)
CL1181.Contig4_All	Integrin	3.59 (up)	8.33 (up)
CL6415.Contig2_All	Cathepsin C	3.49 (up)	7.38 (up)
CL2737.Contig2_All	Ras	3.16 (up)	8.22 (up)
CL2514.Contig2_All	NF-kappa B inhibitor alpha	0.46 (down)	0.19 (down)
Unigene789_All	Clip domain serine proteinase 3	0.47 (down)	0.66 (down)
Unigene31753_All	Tyrosine-protein kinase isoform	0.36 (down)	0.17 (down)
CL3161.Contig1_All	Phosphoinositide 3-kinase isoform	0.49 (down)	0.88 (down)

## Discussion

Transcriptome sequencing is an effective method for obtaining genomic information from non-model organism with no available reference genome. The genomic information is the important basis to discover molecular mechanisms of the organism's biological traits. Due to its superior antiviral immune characteristics, crayfish has become the economically most important aquacultured species in China. However, research on the antiviral molecular mechanisms is scarce. In the present study, *de novo* assembled transcriptomes of crayfish gills were analyzed, and a large number of sequences were obtained. DEGs that differed in expression between normal and WSSV-challenged crayfish gills were studied in detail. All these transcriptomes data are valuable to shed light on the antiviral immune mechanism of crayfish.

So far, several studies based on the NGS technology have reported transcriptome sequencing results for crayfish tissues, including hepatopancreas, muscle, ovary, testis, eyestalk, spermary, epidermis, branchia, intestines, and stomach. Those studies mainly focused on gonadal development (Jiang *et al.*, 2014), neuroendocrinology (Manfrin *et al.*, 2015), and genetic markers (Shen *et al.*, 2014). Transcriptome sequencing results for crayfish antiviral immunity are very limited, and very few works about crayfish transcriptomes changing after virus challenge are reported. So, the aim of the present study was an in-depth analysis of DEGs by functional annotation, orthologous protein clustering, and annotation of signaling pathways related to the immune system to determine the underlying mechanisms involved in the crayfish anti-WSSV immune response.

**Figure 5:**
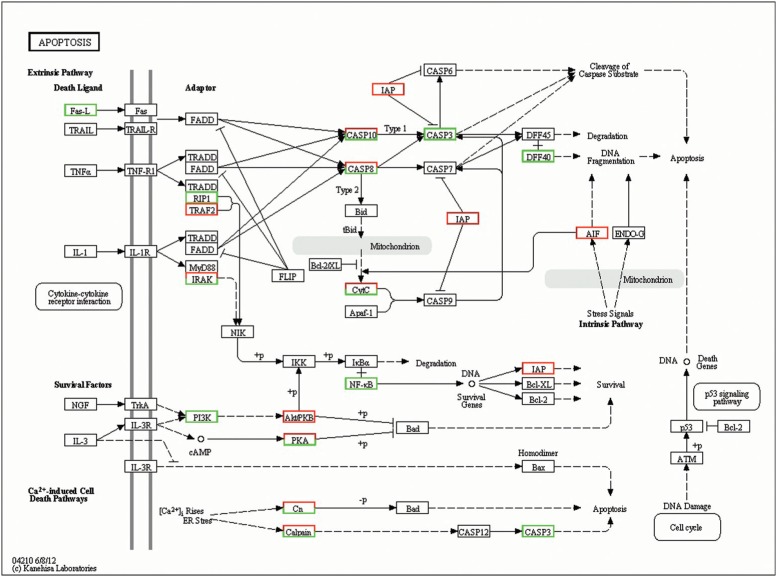
Significant differentially expressed genes (DEGs) identified by KEGG as involved in the apoptosis signaling pathway. Red boxes indicate significantly increased expression, green boxes indicate significantly decreased expression and blue boxes indicate unchanged expression.

Based on the KEGG pathway analysis for DEGs between the GN and GW, some signaling pathways related to the invertebrate innate immune system were identified. Apoptosis is an indispensable physiologic and biochemical process that evolved in eukaryotes to remove excessive and damaged cells to maintain organismal integrity. Apoptosis plays an important role in the processes of cell differentiation, development, and proliferation (Elmore, 2007). When organisms encounter a pathogen infection and other environment stresses, apoptosis can be mobilized to regulate cell metabolism (Igney and Krammer, 2002). Cell proliferation controlled by apoptosis has been reported to be involved in the antiviral immunity response (Du *et al.*, 2013). In the present study, 73 representative innate immune-related genes were significantly differentially expressed in the apoptosis signaling pathway. Within this group, 52 DEGs were significantly up-regulated, and 21 DEGs were significantly down-regulated (Figure 5).

**Figure 6:**
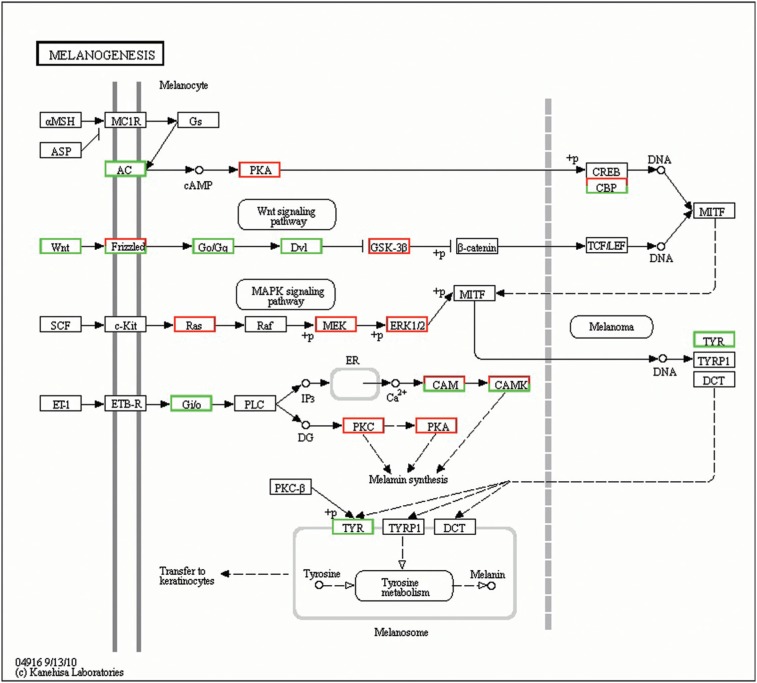
Significant differentially expressed genes (DEGs) identified by KEGG involved in the melanogenesis signaling pathway. Red boxes indicate significantly increased expression, green boxes indicate significantly decreased expression, and black boxes indicate unchanged expression.

Melanogenesis is an important biochemical pathway responsible for melanin synthesis that is controlled by complex regulatory mechanisms (Kim, 2015). Melanin is a vital intermediate in the arthropod humoral immunity response. In the present study, 108 innate immunity-related genes in the melanogenesis pathway were significantly differentially expressed, including 73 significantly up-regulated genes and 35 significantly down-regulated genes (Figure 6).

**Figure 7:**
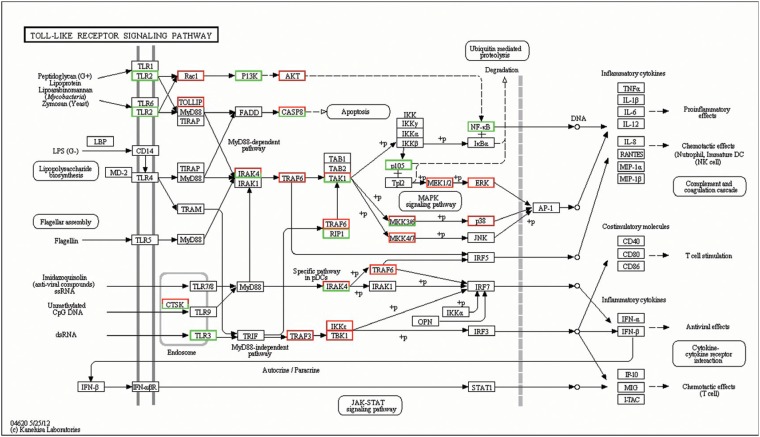
Significant differentially expressed genes (DEGs) identified by KEGG involved in the Toll-like receptors (TLRs) signaling pathway. Red boxes indicate significantly increased expression, green boxes indicate significantly decreased expression, and black boxes indicate unchanged expression.

Toll-like receptors (TLRs) are a group of highly conserved molecules that play a vital role in the recognition of pathogen-associated molecular patterns (PAMPs) and in the activation of innate immune responses to infectious agents (Zhang and Lu, 2015). Many studies have implied the TLRs signaling pathway in both antibacterial and antifungal defense (De Nardo, 2015). In this study, 66 innate immunity-related genes in the TLRs signaling pathway were significantly differentially expressed, including 49 significantly up-regulated genes and 17 significantly down-regulated genes (Figure 7).

The three abovementioned signaling pathways were significantly changed (P < 0.01) between GN and GW, which suggests that they may play an important role in crayfish antiviral immunity responses. These results could provide new insights for future research on crayfish antiviral immunity.

In conclusion, many genes and pathways related to innate immunity were modified after WSSV infection of crayfish gills. Leveraging the gene expression changes into a model of altered network functions could provide new insights into the crayfish antiviral immunity mechanism and could also highlight candidate proteins that should be targeted to solve viral disease problems in the crayfish breeding process.
